# Evaluation of Africa-Europe patient safety hospital partnerships: a framework

**DOI:** 10.1186/1753-6561-5-S6-P321

**Published:** 2011-06-29

**Authors:** PD Rutter, SB Syed, E Kelley

**Affiliations:** 1 WHO Patient Safety, London, UK; 2 WHO Patient Safety, Geneva, Switzerland

## Introduction / objectives

Evaluation is critical to continuous improvement. African Partnerships for Patient Safety (APPS) has 3 core objectives – partnership strength, patient safety improvement and patient safety spread – for which an evaluation framework was necessary. Action on health care-associated infections (HAI) provides a common platform of activity.

## Methods

To mitigate expected challenges to evaluation implementation in African contexts, end-users contributed heavily to framework development, providing contextual awareness and building ownership. Indicators for each of the three objectives were identified, scrutinized and refined. Capture tools were sourced or developed. A three-step process was used: first, expert-led literature review; second, expert consensus; and third, consultative selection of key indicators with end users (African APPS hospitals). Available HAI tools were incorporated into the framework.

## Results

A three-pronged evaluation framework emerged. Six emergent domains of partnership strength (with associated facets for each domain) constituted the basis of evaluating this first core objective. A “3-2-1” approach emerged in evaluating hospital patient safety improvement focused on HAI (3 structure; 2 process; & 1 outcome indicator). An initial 20 measures of spread fell into two broad categories (initial spread activities & ongoing relationships). An evaluation handbook was developed to provide a single source of necessary tools for each of the three objectives.

## Conclusion

We iteratively developed an evaluation framework for a broad-based patient safety programme in a resource-constrained setting. Simplicity was the focus throughout the process. Each of the three framework components can be used separately or in tandem in other settings and programs. Early APPS experiences in framework utilization can guide this.

## Disclosure of interest

None declared.

